# Long-term effects of the Active for Life Year 5 (AFLY5) school-based cluster-randomised controlled trial

**DOI:** 10.1136/bmjopen-2015-010957

**Published:** 2016-11-24

**Authors:** Emma L Anderson, Laura D Howe, Ruth R Kipping, Rona Campbell, Russell Jago, Sian M Noble, Sian Wells, Catherine Chittleborough, Tim J Peters, Debbie A Lawlor

**Affiliations:** 1 School of Social and Community Medicine, University of Bristol, Bristol, UK; 2 MRC Integrative Epidemiology Unit at the University of Bristol, Bristol, UK; 3 Centre for Exercise, Nutrition and Health, School for Policy Studies, University of Bristol, Bristol, UK; 4 School of Public Health, University of Adelaide, Adelaide, South Australia, Australia; 5 School of Clinical Sciences, University of Bristol, Bristol, UK

**Keywords:** children, randomised controlled trial, schools, physical activity, diet

## Abstract

**Objective:**

To investigate the long-term effectiveness of a school-based intervention to improve physical activity and diet in children.

**Design:**

Cluster-randomised controlled trial.

**Setting:**

60 primary schools in the southwest of England.

**Participants:**

Primary school children who were aged 8–9 years at recruitment, 9–10 years during the intervention and 10–11 years at the long-term follow-up assessment.

**Intervention:**

Teacher training, provision of lesson and child–parent interactive homework plans and teaching materials.

**Main outcome measures:**

Primary outcomes were accelerometer-assessed minutes of moderate to vigorous physical activity (MVPA) per day, accelerometer-assessed minutes of sedentary behaviour per day and reported daily consumption of servings of fruit and vegetables.

**Results:**

60 schools with 2221 eligible children were recruited. As in the previously published assessment immediately after the end of the intervention, none of the three primary outcomes differed between children in schools allocated to the intervention, compared with those in control schools at the end of the long-term follow-up (1 year after the end of the intervention). Differences in secondary outcomes were consistent with those at the immediate follow-up, with no evidence that these had diminished over time. Comparing intervention with control schools, the difference in mean child-reported screen viewing at the weekend was −16.03 min (95% CI −32.82 to 0.73), for servings of snacks per day, the difference was −0.11 (95% CI −0.39 to 0.06), in servings of high-energy drinks per day −0.20 (95% CI −0.39 to −0.01) and in servings of high-fat foods per day −0.12 (95% CI −0.39 to 0.00). None of these reached our predefined level of statistical significance, especially after accounting for multiple testing.

**Conclusions:**

School-based curriculum interventions alone are unlikely to have a major public health impact on children's diet and physical activity.

**Trial registration number:**

ISRCTN50133740, Post-results.

**Strengths and limitations of this study**

- The study was designed to take account of known sources of bias in other randomised controlled trials in this area, with concealed random allocation of participants, outcome assessors who were blinded to which group the schools and children had been randomised to and objective measurements of physical activity and sedentary behaviour with accelerometers.
- Our sample size calculation took account of the likely degree of clustering within schools.
- The study was undertaken in state schools in the southwest of England that covered a range of deprivation levels and urban and rural communities, but results may not be generalisable to more ethnically diverse populations in the UK or beyond the UK.
- There were missing data for the accelerometer assessed outcomes, but a range of sensitivity analyses did not alter our findings and levels of weartime and valid accelerometer data were similar in the intervention and control arms.

## Introduction

Low levels of physical activity and fruit and vegetable consumption in childhood track into adulthood^1–3^ and are associated with greater adiposity, adverse cardiometabolic risk factors, behavioural problems, low mood and poorer academic attainment.^1–7^ School-based interventions have the potential to efficiently change behaviours to healthier levels, or delay age-related changes in behaviour,^8^ since most children attend school. However, previous randomised controlled trials (RCTs) of such interventions have potentially important sources of bias and few have explored long-term outcomes beyond the end of the intervention period.

A systematic review and meta-analysis of 44 school-based RCTs found beneficial effects on moderate or vigorous physical activity (MVPA) during school hours, but the authors noted that benefit might have been exaggerated due to the outcome assessment being self-reported/parental-reported and not blind to school allocation in most trials and because of the marked loss to follow-up in several trials.^9^ In many of those RCTs, the intervention included extra compulsory physical activity lessons or activities during school break-times. Those have the advantage that they do not interrupt the school curriculum, but in the absence of any long-term follow-up beyond the intervention period, it is impossible to determine whether the greater time spent in MVPA is simply as a result of a level of compulsion to be more active. Evidence from observational epidemiological studies suggests that compulsory physical activity in lessons or break-time in school are associated with more school-based activity, but not with more activity outside of school or if the activity stops being compulsory.^10^ ^11^ A systematic review restricted to studies that had used objectively assessed activity using accelerometers and did not restrict the outcome to activity during school hours found some evidence of benefit of a similar magnitude in family-focused and school curriculum interventions, but noted that the magnitude of effect was modest.^12^ Reviews of interventions to reduce time spent in sedentary behaviour have similarly noted some evidence of effect, but cautioned about likely sources of bias, including lack of adequate concealment of random allocation, subjective outcome measurements with no blinding of participants and little evaluation that effects were sustained long-term postintervention.^13^ ^14^ Likewise, two systematic reviews of school-based interventions to increase fruit and vegetable consumption found some possible evidence of modest effect but were concerned about lack of adequate concealment of random allocation and failure to take account of clustering within analyses.^15^ ^16^

The Active for Life Year 5 (AFLY5) study^17^ was a large school-based cluster RCT. It was designed to address many of the limitations that had been identified in previous RCTs of interventions to improve physical activity and diet in children^9–16^ by objectively measuring physical activity and sedentary behaviour and by determining effects on outcomes immediately after the end of the intervention and 12 months later. At the end of the intervention period (immediate follow-up), the intervention was ineffective at improving any of the three primary outcomes (time spent in moderate to vigorous physical activity, time spent in sedentary activity and fruit and vegetable consumption); however, it did result in improvements in three of the nine secondary outcomes (child-reported time spent screen-viewing at weekends, consumption of snacks and consumption of high-energy drinks).^18^ A cluster RCT design was necessary, given the intervention is at the level of schools (rather than individual children).

In this paper, we report the long-term effects of the intervention on the primary and secondary outcomes that were assessed ∼12 months postintervention. Our initial aim when designing the study was to be able to determine whether any effects of the intervention would last beyond the period of the intervention. Given we now know the immediate postintervention results,^18^ our aim in this paper was to determine whether any effects on primary outcomes emerged at the 12-month follow-up assessment (ie, whether there was a delayed effect of the intervention on the primary outcomes) and whether effects on secondary outcomes that were observed immediately after the intervention were maintained, decreased or increased 12 months after the intervention. In this and the previous paper, the intervention is delivered at the cluster (school) level and outcomes are measured and analysed on individual children, with the clustering appropriately taken account of in the statistical analyses.

## Methods

### Study design and participants

AFLY5 was a school-based, cluster RCT. Clustering was at the level of the schools, with eligibility for study entry being: (1) any state primary or junior schools that (2) provided education to children aged 8–11 years and (3) were within the Bristol City and North Somerset administrative areas (both areas in the southwest of England). All children in UK school year 4 (age 8–9 years) at the time of recruitment were eligible for recruitment if their parents provided consent and they assented (see below).

A total of 60 state primary and junior schools were recruited between March and July 2011: 46 in Bristol and 14 in North Somerset, southwest England. At the time of recruitment, participants were aged 8–9. Full details of the trial have been published previously, so only a brief summary will be given here.^17–19^ The trial was registered prior to recruitment of schools or data collection (http://www.controlled-trials.com/ISRCTN50133740). Analyses have been undertaken in accordance with a published analytical plan that was approved by the Trial Steering Committee.^17–19^

### Ethical approval and consent

Ethical approval was obtained from the University of Bristol Faculty of Medicine and Dentistry Committee for Ethics (reference number 101115). Parents/guardians of children in Year 4 were sent a letter and information sheet about the study, with an opt-out consent form for each of the measurements and the opportunity to contact the research team to discuss the study as well as information about being able to withdraw at any stage. An information sheet for the child was sent at the same time that the letter was sent to the parents. Children were given a second copy of this information sheet at the time that measurements were undertaken and they were asked to give signed assent to each of the measurements.

### Randomisation

Schools were defined as having high or low involvement in any initiatives aimed at increasing physical activity, reducing sedentary behaviour or increasing fruit and vegetable consumption, based on their report of involvement in local or national initiatives. Schools were also split into tertiles based on their score on the English Index of Multiple Deprivation 2010 (IMD 2010).^20^ Schools were grouped into six mutually exclusive strata by these two characteristics and randomly allocated to control or intervention within these strata.^17–19^ Randomisation was undertaken by DAL who was unaware of any other characteristics of the schools. School was concealed using the Bristol Randomised Trials Collaboration's automated (remote) system. After randomisation, one school refused to undertake the intervention; the head reported that they had hoped they would be randomised to control and did not have the time or capacity to accommodate the intervention. This school was retained in the relevant analyses on an intention-to-treat basis.

### Intervention

The intervention was adapted from a previously evaluated US intervention^21^ and is based on Social Cognitive Theory,^22^ with a particular emphasis on increasing the children's self-efficacy (perceived competence) to be physically active and eat a healthy diet.^23^ Full details of the trial intervention have been published in the trial protocol and the paper reporting the immediate effect of the intervention.^17^ ^18^ It comprised:

1. Training for classroom teachers and learning support assistants, provided by the trial manager, a nutritionist and physical education specialist. The training took place over a whole day (8–9 hours) in a non-school location and where the teachers/learning support assistants and those delivering the training would not be interrupted. Teachers/learning support assistants were given a choice of days to attend the training and schools were financially compensated for the cost of replacement teachers while their staff attended training. At the training days, the rationale for the intervention was explained and each lesson and homework activity was discussed and then taught in interactive ways. Time was provided for questions and discussion. Teachers were instructed to deliver 16 lessons, 10 of which had associated homework. They were told that they could adapt the teaching plans and materials, as they would with other lessons, for example, to suit their own style and the range of abilities in their class, but the aims and knowledge/skills to be imparted should not be changed.
2. Provision of 16 lesson plans and teaching materials, including pictures, CDs and journals for teachers or learning support assistants to deliver over two out of the three school-terms (6–7 months). The 16 lessons included 9 that were primarily related to how to be more active and less sedentary and why this was important, 6 to healthy nutrition and how to achieve this and 1 about reducing screen viewing. Each lesson did, however, combine different aspects of healthy behaviour. For example, in the physical activity lessons, the children played games based on the food groups using photographs of food which reinforced the content of the nutrition lessons. Similarly, in the lesson (and associated homework) for reducing screen-viewing (called ‘Freeze my TV’), children were taught how to replace regular television watching with active play on some days.
3. Provision of 10 parent–child interaction homework activities. The activities were designed to involve parents and other family members in the behaviour change process and reinforced the messages delivered during lessons. The homeworks included activities such as: ‘Freeze my TV’, in which a specific time that would normally be spent watching television would be replaced with physically active play involving the parents and other family members that the child would write a log about; cooking simple healthy food at home; playing ‘Top Grubs’ a card game based on trumps with pictures of food, such that higher scoring (trumping) foods are the healthier ones; and measuring the sugar content of drinks that the family have at home or include in school/work lunch packs.
4. Information was provided for schools to insert (as they wished) in their school newsletters about the importance of increasing physical activity, reducing sedentary behaviour and improving diet. The inserts were sent to all intervention schools on three occasions over the period of the intervention. Schools were free to edit these and insert none, all or some of them.
5. Written information for parents on how to encourage their children to eat healthily and be active was delivered via the school children at the start of the intervention.

The intervention took place when the children were aged 9–10 years (in UK school Year 5) after baseline assessment. Schools randomised to the control group continued standard education provision for the school year, and any involvement in additional health-promoting activities, but had no access to the intervention teacher training or the teaching materials.

### Outcomes

Box 1 lists the three primary and nine secondary outcomes.

Box 1 **AFLY5 primary and secondary outcomes**

Primary outcomes

Accelerometer-assessed mean time per day spent doing moderate/vigorous physical activity MVPA (min/day)

Accelerometer-assessed mean time per day spent in sedentary activity (min/day)

Self-reported (validated questionnaire) servings of fruit and vegetables consumed per day (servings per day; treated in all analyses as a continuous variable)

Secondary outcomes

Self-reported (validated questionnaire) mean time spent screen viewing on a typical weekday (min)

Self-reported (validated questionnaire) mean time spent screen viewing on a typical weekend day (min)

Self-reported (validated questionnaire) servings of snacks consumed per day (servings per day; treated in all analyses as a continuous variable)

Self-reported (validated questionnaire) servings of high-fat foods consumed per day (servings per day; treated in all analyses as a continuous variable)

Self-reported (validated questionnaire) servings of high-energy drinks consumed per day (servings per day; treated in all analyses as a continuous variable)

Body mass index determined from weight and height measured in classrooms by two study fieldworkers (kg/m^2^; treated in all analyses as an SD z-score)

Waist circumference measured in classrooms by two study fieldworkers (mm; treated in all analyses as an SD z-score)

General overweight/obesity, determined by the International Obesity Task Force thresholds of body mass index for children (taking account of their age and sex) (binary outcome)

Central overweight/obesity determined by thresholds of UK age-specific and sex-specific reference charts for waist circumference and defined by the International Diabetes Federation (binary outcome)

### Participant assessments

Baseline assessment (prior to intervention) was undertaken either between April and June 2011 or between September and November 2011, when the children were aged 8–9 years (ie, before and after the school summer break). Immediate follow-up assessment was completed immediately postintervention ∼12 months after the baseline assessment and the long-term assessment (with which this paper is concerned) took place 12 months after the immediate assessment, during which time the children were not exposed to the intervention. Every attempt was made to undertake the assessments in the same order, so that the seasons would be similar at each assessment time.

Assessments measured primary and secondary outcomes, together with demographic characteristics and were conducted identically at each time point following published protocols.^17^ ^19^ They were completed by trained fieldworkers who were blinded as to which arm of the trial schools had been allocated. Full details of these assessments have been published previously^17^ ^19^ and are summarised here. Questionnaires asked for information on dietary intake and screen-time viewing and other characteristics and were administered in the classroom with at least one fieldworker present. Weight, height and waist circumference were measured in a private room by one of the trained fieldworkers, with a second fieldworker present in the room. All fieldworkers had passed Criminal Records Bureau checks, as required for working with children at the time that these data were collected. Physical activity was assessed using ActiGraph GT3X+ accelerometers (Actigraph LLC, Pensacola, Florida, USA) and time spent per day being sedentary and in moderate to vigorous activity were calculated using standard protocols as described previously.^17^ ^19^

### Sample size calculation and account of multiple testing

Sample size calculations indicated that for the three primary outcome and nine secondary outcome measurements (including taking account of multiple testing with the secondary outcomes), a total of 60 schools with 1500 pupils (750 in each arm) needed to be recruited, so that 1275 (allowing for loss to follow-up) pupils could be included in the analyses.^17^ This number provided adequate power to detect what we considered to be minimally important effects.^17^ ^19^ We recruited 60 schools and a total of 2221 pupils, and included between 1066 and 2052 pupils in our analyses for different outcomes. Analyses for accelerometer-based outcomes were on fewer participants than our sample size calculation suggested (N=1066) because of a large proportion of participants not returning or not wearing the accelerometer for at least 8 hours for 3 days, the minimum required to be included in the study.^17^ ^19^

### Statistical analyses

Full details of the analysis plan have been published previously.^19^ Briefly, main analyses assessing the effect of the intervention on the primary and secondary 12 months postintervention were conducted as intention-to-treat, with missing data at baseline being replaced with a value of 999 and a variable to indicate missing data at baseline (0=not missing, 1=missing) being included in regression models, as recommended by White *et al*.^24–26^ For primary outcomes, the level of statistical significance used was p<0.05 and for secondary outcomes, the level of statistical significance used was p<0.01, after correcting for multiple testing.^19^ A series of sensitivity analyses were conducted to test assumptions regarding the nature of missing data at baseline and at each of the follow-up assessments (see detailed analysis plan^19^ for discussion of these assumptions and the sensitivity analyses). Multilevel regression models were used to account for clustering (non-independence) of children within schools.^19^ All analyses included adjustment for the following baseline variables: age, sex, baseline measure of the outcome being analysed, involvement in other healthy behaviour promoting activities and school level deprivation. A secondary per-protocol analysis was undertaken, in which classes in the intervention arm were only included in analyses if teachers had taught at least 70% (11 of 16) of the AFLY5 lessons. There was one school for which we were unable to confirm how many lessons had been taught. For that school, we first did analyses assuming that they had been taught at least 11 lessons and then repeated them assuming that they had been taught fewer than 11; the results were identical whichever of these alternatives were used. We additionally assessed whether the effect of the intervention on accelerometer-assessed outcomes differed by week or weekend day and whether the results were affected by implausible values as defined previously. The researchers undertaking the analyses were blinded to (unaware of) whether schools had been allocated to intervention or control arms.

As detailed in the published statistical protocol,^19^ we initially planned to assess change in outcomes between baseline and the long-term follow-up using multilevel models to estimate a trajectory of the repeat measurements (baseline, immediate follow-up, long-term follow-up) within each individual, with random effects to quantify the estimated person-specific deviation from the study mean in terms of the intercept (baseline measurement) and rate of change (slope). However, when we attempted to run these models, they did not converge. This is likely because there were only three measurement occasions, meaning that the model did not have sufficient df. Therefore, we conducted analyses at a single time point as described above (ie, assessed the effect of the intervention on outcomes at the long-term follow-up) and plotted differences between the randomised groups at each time point in order to illustrate any notable changes in estimates of the primary and secondary outcomes between baseline and immediate and long-term follow-up.

## Results

**Figure 1:**
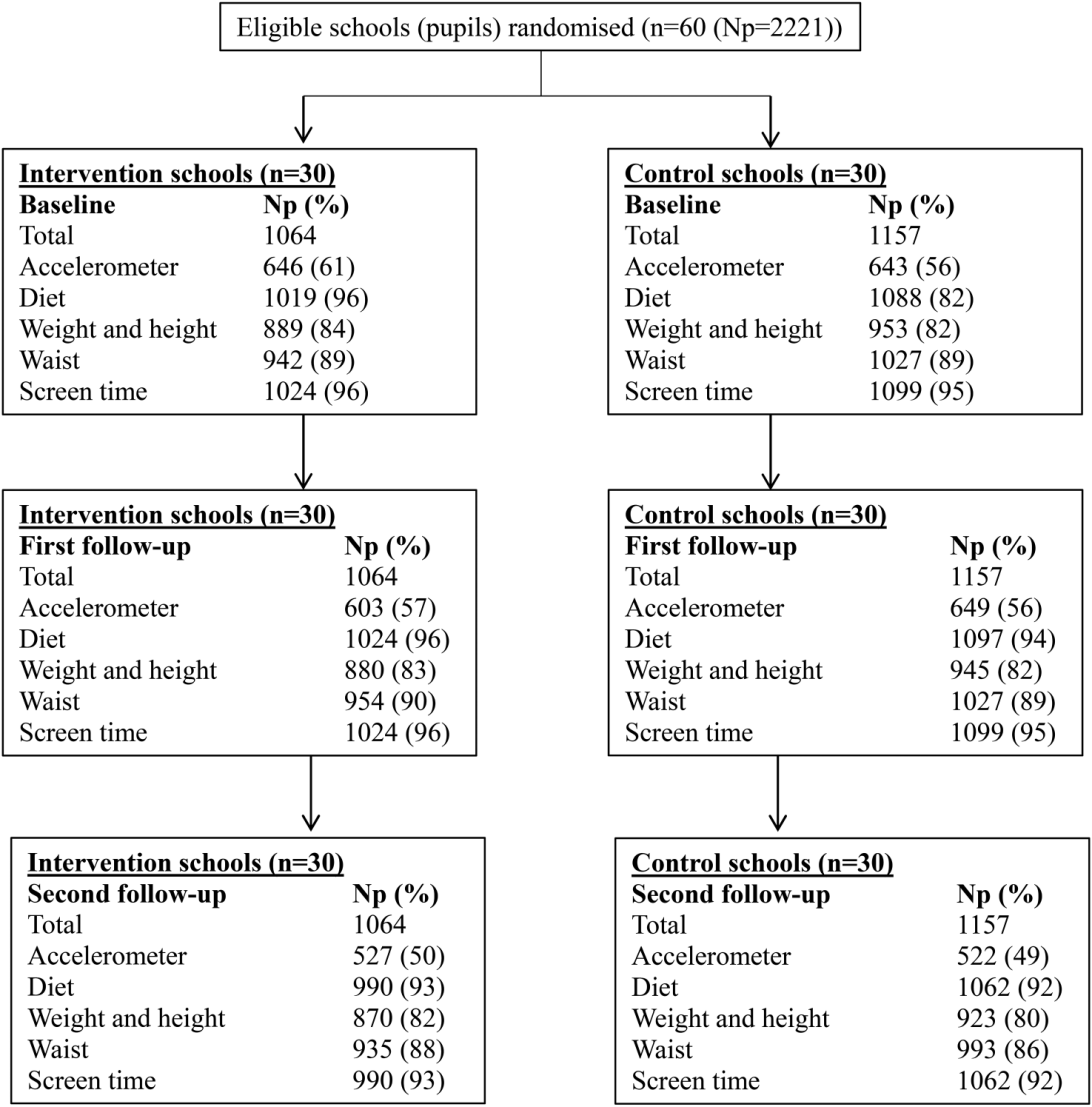
Trial profile. Np, number of participants (school pupils). No schools withdrew from the study, so all randomised units are present at baseline and at both follow-up assessments. Percentages for proportions of children with each measurement at baseline and at follow-ups are of total number of children who were pupils in randomised schools at baseline. Not all pupils with follow-up measures necessarily had data on the same measure at baseline (or vice versa), because of different pupils being absent at baseline and follow-up assessments at each time point, and because of pupils leaving or moving between schools. In all analyses, study participants were analysed in the group (intervention or control) to which they were randomised.

Figure 1 shows the trial profile. Of the 2242 potentially eligible children in the 60 participating schools, 10 left the school prior to randomisation and baseline data collection and for 11, their parents or carers did not provide consent to participate in any aspect of the study. All other children (N=2221; 1064 in the schools that were randomised to intervention and 1157 in those randomised to control schools), irrespective of whether or not we have all the data for them, are included in the analyses presented here (with numbers differing for each outcome in the main analyses as a result of some missing data). Proportions with data for each outcome were similar in intervention and control schools at baseline and at the second follow-up assessment at 12 months postintervention (figure 1). Baseline characteristics were similar between children in intervention schools and those in control schools (table 1).

**Table 1:** Comparison of baseline characteristics by randomised group

	Unit and type of summary measure	Intervention schools Number of participants=1064 Number of schools=30		Control schools Number of participants=1157 Number of schools=30	
Characteristic		Number	Distribution	Number	Distribution
Age	Mean (SD) years	1024	9.5 (0.3)	1099	9.5 (0.3)
MVPA*	Mean (SD) min	912	59 (23)	928	56 (21)
Sedentary behaviour*	Mean (SD) min	912	422 (72)	928	416 (68)
Servings of fruit and vegetables	Median (IQR) number/day	1019	1 (0 to 2)	1088	1 (0 to 2)
Servings of snacks	Median (IQR) number/day	1019	2 (1 to 3)	1088	2 (1 to 3)
Servings of high-fat foods	Median (IQR) number/day	1019	0 (0 to 1)	1088	1 (0 to 1)
Servings of high-energy drinks	Median (IQR) number/day	1019	2 (1 to 3)	1088	2 (1 to 3)
BMI	Mean (SD) z-score	889	−0.06 (0.94)	953	0.05 (1.04)
WC	Mean (SD) z-score	942	−0.03 (0.97)	1027	0.03 (1.02)
Screen-viewing weekday	Median (IQR) min	1024	105 (45 to 240)	1099	105 (45 to 225)
Screen-viewing Saturday	Median (IQR) min	1024	90 (30 to 240)	1099	105 (30 to 240)
Total number of valid days of wearing accelerometer†	Median (IQR) days	912	3 (2 to 5)	928	3 (2 to 4)
Total number of valid weekdays of wearing accelerometer†	Median (IQR) days	979	2 (2 to 3)	1025	2 (1 to 3)
Total hours of wearing accelerometer on valid days*	Mean (SD) hours/day	912	11.6 (1.5)	928	11.5 (1.4)
Hours of wearing accelerometer on valid weekdays†	Mean (SD) hours/day	896	11.8 (1.6)	919	11.7 (1.5)
*Categorical variables*					
Gender	N (%) female	520	49%	608	52%
	N (%) male	544	51%	549	48%
General overweight/obesity	N (%) No	717	81%	743	78%
	N (%) Yes	172	19%	210	22%
Central overweight/obesity	N (%) No	601	64%	631	61%
	N (%) Yes	341	36%	396	39%
Returned accelerometer	N (%) No	85	8%	132	11%
	N (%) Yes	979	92%	1025	89%
Wore accelerometer for requested amount of time	N (%) No	820	77%	953	82%
	N (%) Yes	244	23%	204	18%
Wore accelerometer for required amount of time	N (%) No	418	39%	514	44%
	N (%) Yes	646	61%	643	56%
School involved in other health-promoting activities	N (%) No	264	25%	446	39%
	N (%) Yes	800	75%	711	61%
School deprivation score	N (%) low	315	30%	460	40%
	N (%) medium	368	35%	345	30%
	N (%) high	381	36%	352	30%

Note some % within categories do not sum to exactly 100 because of rounding.

*Including only participants with at least 3 days of valid data.

†Including all valid days, regardless of the number of valid days.

BMI, body mass index; MVPA, moderate or vigorous physical activity; WC, waist circumference.

**Figure 2:**
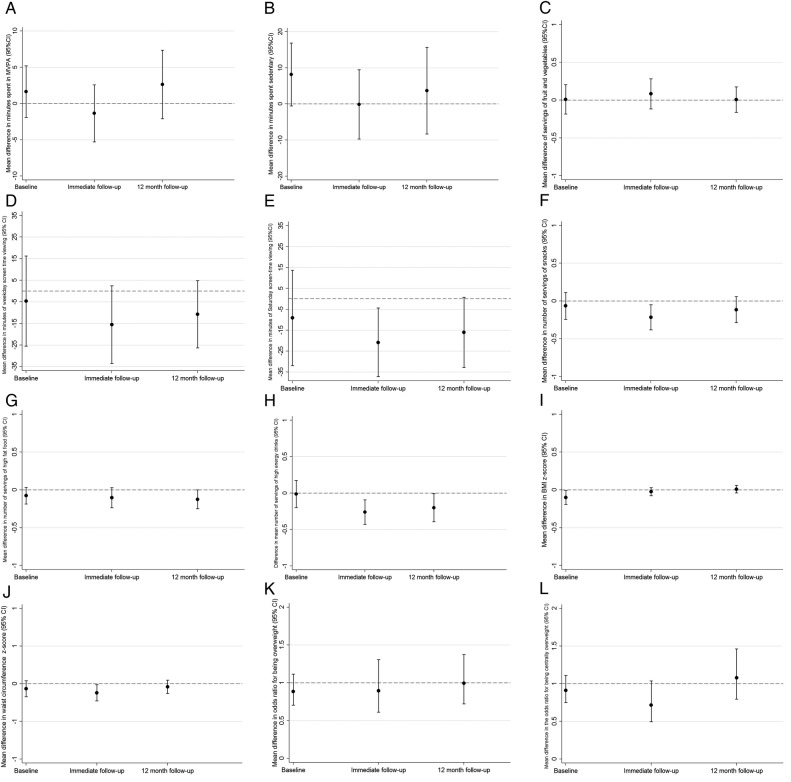
Difference in means and ORs for the intervention compared with the control group for the three primary outcomes and nine secondary outcomes, assessed at baseline, first follow-up (conducted immediately after the end of the intervention) and second follow-up (12 months postintervention). (A) Accelerometer-assessed time spent in moderate to vigorous physical activity. (B) Time spent in sedentary behaviour. (C) Servings of fruit and vegetables per day. (D) Time spent screen viewing on weekdays. (E) Time spent screen viewing on Saturdays. (F) Servings of snacks per day. (G) Servings of high-fat foods per day. (H) Servings of high-energy drinks per day. (I) Body mass index z-score (as a continuous variable). (J) Waist circumference z-score (as a continuous variable). (K) General overweight or obesity (based on BMI measurements). (I) Central overweight/obesity based on waist circumference measurements. The figures all show differences in means for continuous variables (graphs A–J) and ORs for binary outcomes (graphs K and L), comparing those in the intervention arm of the trial with those in the control arm (dots), together with 95% CIs (vertical lines with horizontal caps representing the limits). The dashed horizontal lines represent the null values (zero for all differences in means of continuous variables and one for ORs of binary outcomes).

Figure 2A–L shows differences in means or ORs between the control and intervention group for the three primary and nine secondary outcomes at baseline, immediate follow-up and long-term (12 months) follow-up. These show that differences in means (and ORs for general and central overweight/obesity) between children in intervention and control schools were essentially the same at this long-term follow-up as they were immediately after the intervention, when examining point estimates. Differences in the primary outcomes were consistent with the null hypothesis (figure 2A–C). Differences in secondary outcomes were consistent with those seen at the end of the immediate follow-up (figure 2D–L), with no evidence that the previously reported beneficial effects for child-reported screen viewing at weekends (figure 2E), consumption of snacks (figure 2F) and consumption of high-energy drinks (figure 2H) had notably diminished (or increased) in magnitude over time (figure 2). However, there was no strong statistical support for any effect of the intervention on primary and secondary outcomes at 12 months after the intervention. Table 2 shows differences in means or ORs for all outcomes at the long-term follow-up from the main intention-to-treat analyses. None of the three primary outcomes differed, nor the nine secondary outcomes, reached our predefined level of statistical significance for an effect after accounting for multiple testing.

**Table 2:** Main intention-to-treat analyses of the effect of AFLY5 intervention on primary and secondary outcomes assessed 12 months postintervention

	Control group (reference group)		Intervention group		Main comparison between the two groups (intervention vs control)		
Outcome (primary/secondary)	Np	Mean (SD) or number (%)	Np	Mean (SD) or number (%)	Np	Difference in means or OR (95% CI)	p Value
Continuous outcomes							
** Time spent in MVPA (min/day)**	522	52.56 (20.67)	527	54.37 (22.23)	1049	2.48 (−1.80 to 6.77)	0.26
** Time spent in sedentary behaviour (min/day)**	522	461.78 (66.33)	527	465.46 (70.61)	1049	2.79 (−7.78 to 13.37)	0.60
** Servings of fruit and vegetables (number/day)**	1062	1.80 (1.55)	990	1.82 (1.59)	2052	0.01 (−06 to 0.17)	0.94
Time spent screen-viewing (min/day weekday)	1062	148.01 (126.39)	990	138.88 (125.00)	2052	−10.74 (−26.30 to 4.81)	0.18
Time spent screen-viewing (min/day Saturday)	1062	180.52 (164.82)	990	167.71 (156.28)	2052	−16.03 (−32.82 to 0.73)	0.06
Body mass index (z-score)	923	0.03 (1.02)	870	−0.03 (0.97)	1793	0.01 (−0.04 to 0.06)	0.72
Waist circumference (z-score)	993	0.03 (1.04)	935	−0.03 (0.95)	1928	−0.04 (−0.13 to 0.05)	0.36
Servings of snacks (number/day)	1062	2.11 (1.55)	990	1.99 (1.47)	2052	−0.11 (−0.29 to 0.06)	0.19
Servings of high-fat foods (number/day)	1062	0.86 (0.94)	990	0.74 (1.07)	2052	−0.12 (−0.25 to 0.00)	0.05
Servings of high-energy drinks (number/day)	1062	2.38 (1.58)	990	2.19 (1.45)	2052	−0.20 (−0.39 to −0.01)	0.04
Binary outcomes							
Generally overweight/obese	923	194 (21.02)	870	175 (20.11)	1793	1.00 (0.72 to 1.37)	0.98
Centrally overweight/obese	993	421 (42.40)	935	394 (42.14)	1928	1.08 (0.80 to 1.46)	0.62

Numbers of participants vary by outcome as indicated in the table.

Outcomes in bold are primary outcomes (p<0.05 indicates statistical significance); all others are secondary outcomes (p<0.01 indicates statistical significance after taking account of multiple testing).

All differences in means/ORs with their 95% CIs have been estimated using a multilevel model to account for clustering (non-independence) among children from the same school. Multilevel multivariable linear regression was used for effects of the intervention on continuously measured outcomes and multilevel multivariable logistic regression was used for binary outcomes.

The following baseline/school stratifying variables were included: age, gender, the baseline measure of the outcome under consideration, school involvement in other health-promoting behaviours, school area level deprivation.

In these analyses, participants were included for each outcome if they had a follow-up measurement of that outcome; for missing baseline data, we used an indicator variable as described by White and Thompson,^21^ which means for each outcome, participants are included even if they do not have a baseline measurement.

MVPA, moderate to vigorous physical activity (accelerometer assessed); Np, number of participants.

Results from the per-protocol analyses were consistent with the intention-to-treat analyses results (table 3). Results were similar in all sensitivity analyses applying different assumptions about missing data (see online supplementary tables S1–S4). Results were also similar when we looked separately at time spent in MVPA and time spent in sedentary behaviour by weekday and weekend (see online supplementary table S5).

10.1136/bmjopen-2015-010957.supp1 supplementary tables

**Table 3:** Per-protocol analyses of the effect of AFLY5 intervention on primary and secondary outcomes assessed 12 months postintervention

	Control group (reference group)		Intervention group		Main comparison between the two groups (intervention vs control)		
Outcome (primary/secondary)	Np	Mean (SD) or number (%)	Np	Mean (SD) or number (%)	Np	Difference in means or OR (95% CI)	p Value
Continuous outcomes							
** Time spent in MVPA (min/day)**	522	52.56 (20.67)	356	54.15 (22.27)	878	2.63 (−2.10 to 7.37)	0.28
** Time spent in sedentary behaviour (min/day)**	522	461.78 (66.33)	356	466.17 (70.58)	878	3.67 (−8.32 to 15.66)	0.55
** Servings of fruit and vegetables (number/day)**	1062	1.80 (1.55)	701	1.91 (1.66)	1762	0.05 (−0.15 to 0.25)	0.63
Time spent screen-viewing (min/day weekday)	1062	148.01 (126.39)	701	134.98 (120.94)	1762	−8.97 (−26.81 to 8.87)	0.32
Time spent screen-viewing (min/day Saturday)	1062	180.52 (164.82)	701	159.35 (149.97)	1762	−21.73 (−41.19 to −2.26)	0.03
Body mass index (z-score)	923	0.03 (1.02)	612	−0.03 (0.98)	1535	0.01 (−0.05 to 0.07)	0.69
Waist circumference (z-score)	993	0.03 (1.04)	657	−0.04 (0.94)	1650	−0.03 (−0.13 to 0.06)	0.52
Servings of snacks (number per day)	1062	2.11 (1.55)	701	2.07 (1.48)	1762	−0.03 (−0.23 to 0.16)	0.72
Servings of high-fat foods (number per day)	1062	0.86 (0.94)	701	0.75 (1.15)	1762	−0.11 (−0.26 to 0.04)	0.14
Servings of high-energy drinks (number per day)	1062	2.38 (1.58)	701	2.22 (1.43)	1762	−0.18 (−0.41 to 0.5)	0.12
Binary outcomes							
Generally overweight/obese	923	194 (21.02)	612	121 (19.77)	1535	0.98 (0.68 to 1.41)	0.91
Centrally overweight/obese	993	421 (42.40)	657	272 (41.40)	1650	1.06 (0.76 to 1.49)	0.72

Numbers vary by outcome as indicated in the table.

Per-protocol analysis defined as teaching at least 70% (11 out of the 16) AFLY5 lessons. All participants from the intervention schools where the teacher taught fewer than 11 lessons are excluded from these analyses.

Outcomes in bold are primary outcomes (p<0.05 indicates statistical significance); all others are secondary outcomes (p<0.01 indicates statistical significance after taking account of multiple testing).

All differences in means/ORs with their 95% CI have been estimated using a multilevel model to account for clustering (non-independence) among children from the same school. Multilevel multivariable linear regression was used for effects of the intervention on continuously measured outcomes and multilevel multivariable logistic regression was used for binary outcomes.

The following baseline/school stratifying variables were included: age, gender, the baseline measure of the outcome under consideration, school involvement in other health-promoting behaviours, school area level deprivation.

In these analyses, after removal of schools that did not teach at least 11 out of 16 of the lessons, participants were only included for each outcome if they had a follow-up measurement of that outcome. For partial missing baseline data, we used an indicator variable as described by White and Thompson,^21^ which means for each outcome participants are included even if they do not have a baseline measurement.

MVPA, moderate to vigorous physical activity (accelerometer assessed); Np, number of participants.

## Discussion

In this school-based cluster RCT, aimed at increasing physical activity, reducing sedentary behaviours and improving diet in school aged children, we found results at 12 months after the intervention had ended (ie, with no further lessons or teaching aimed at promoting healthy activity and dietary levels during that 12 months) were essentially the same as those seen immediately after the end of the intervention in terms of size of effect. The lack of any effect on the three primary outcomes—time spent in MVPA, time spent in sedentary behaviour and fruit and vegetable consumption—was still observed 12 months later and the beneficial effects on three secondary outcomes (reported screen-viewing at weekends, consumption of snacks and of high-energy drinks) were still somewhat present at 12 months postintervention. However, slight attenuation of the effect on these secondary outcomes meant that at this long-term follow-up, none of our outcomes (primary or secondary) reached our prespecified level of statistical significance.

### Meaning of study findings

While the effects for these secondary outcomes were consistent in magnitude with those seen at the immediate follow-up, they did not reach our prespecified level of statistical significance. Thus, these results suggest that apparent benefits on these secondary outcomes are due to chance.

As discussed in our previous publication of effects immediately at the end of the intervention,^18^ the lack of effect on primary outcomes, in particular on the objectively assessed accelerometer outcomes, might highlight the importance of societal and structural changes to support greater levels of activity, over and above any intervention at a school level.^18^ Our intervention was based on theory,^22^ ^23^ built on a similar intervention that had been previously shown to work in the USA^21^ and in pilot work, conducted by us, it was shown to fit well with the primary school national curriculum in the UK.^27^ Furthermore, the detailed process evaluation conducted as part of the full AFLY5 RCT, in which we used quantitative measures of intervention delivery and qualitative focus groups with children and indepth interviews with teachers and parents,^28^ showed that on average 77% of the intervention lessons and homeworks were delivered and reached 95% of the children in intervention schools. However, teachers felt lack of time and the need to prioritise numeracy and literacy skills over the health-promoting lessons of our intervention were important barriers to them and the children being more fully engaged with AFLY5.^28^ The process evaluation also highlighted that in general, teachers did not like teaching physical activity, and had a tendency to delegate such lessons to teaching assistants. This might also have contributed to the null effects, particularly for the activity outcomes. Finally, our process evaluation suggests that in the context of rapidly developing technologies, the time taken to develop, test the feasibility of, and pilot, school-based interventions before completing large scale RCTs, as we have performed in AFLY5, may mean that by the time school-based interventions get to the full-scale RCT, the intervention is being implemented with out-of-date methods of delivery.^28^ ^29^

While using schools for universal promotion of healthy behaviours is appealing, a key implication of our findings is that this alone is unlikely to have benefit. Pressures on schools to deliver academic success and the fact that teachers do not necessarily feel equipped, responsible for, or in the case of physical activity, enjoy promoting health behaviours,^28^ suggest that curriculum-based health promotion alone is unlikely to benefit population health. Our RCT was large and well conducted and the results suggest that further investment in RCTs of curriculum-based interventions (alone) to improve children's diet and activity are not wanted. Whether investing in extracurricular activities, including in the necessary human resources (eg, people who are appropriately trained and skilled), structural resources (appropriate space) and equipment, would be beneficial at a population level is unclear and may warrant further evaluation. Societal interventions such as those that were envisaged as a legacy of the 2012 Olympics, and the more recent ‘sugar tax’ may be beneficial but will require a natural experiment type approach,^30^ rather than an RCT, for their evaluation. Evaluation of past major sporting events and early assessments of the 2012 Olympics suggest that like our assessment of a school-based curriculum, much more intense, comprehensive (across all levels of society—home, neighbourhoods, schools, work, government, transport systems) and long-term investments are required to support the next generation to be more active and eat healthier.^31–33^

### Strengths and limitations

The study was designed to take account of known sources of bias in other RCTs in this area. A protocol was published before recruitment started, and a detailed analysis plan was written before any access to the study data. We developed an intervention according to guidelines for complex interventions, with the theoretical rationale for the intervention described in detail elsewhere.^18^ Our sample size calculation, which took account of the likely degree of clustering within schools, indicated that we needed a total of 1275 children to be included in the analyses. For all outcomes, except those related to accelerometer data, we achieved considerably higher numbers than this target. The number included in the main analyses for accelerometer-based data was somewhat smaller than this at 1066. Sample size calculations are an approximation of the numbers needed, and we doubt that such a small difference will have had a major effect on our conclusions. Furthermore, wear time was similar in children in intervention and control schools; moreover, in sensitivity analyses using different approaches to dealing with missing data and which included 2052 children even for the accelerometer outcomes, the results were essentially the same as in the main analysis. One school refused to deliver any of the intervention, and others did not deliver all of the lessons. However, the per-protocol analysis, which did not differ from the main intention-to-treat analysis, shows that this does not explain the null results.

## Conclusion

This long-term follow-up of a large well-conducted school-based RCT has found similar results to those found immediately after the intervention period. None of the primary or secondary outcomes reached our predefined levels of statistical significance, suggesting that apparent benefits on some secondary outcomes are due to chance. Overall, together with our process evaluation, these findings suggest that curriculum-based interventions alone are unlikely to make a major impact on promoting healthy levels of physical activity and healthy diets in primary school children.
