# Supplementary material for: Long-term effects of the Active for Life Year 5 (AFLY5) school-based cluster-randomised controlled trial
Source: BMJ Open. 2016 Nov 24;6(11):e010957. doi: 10.1136/bmjopen-2015-010957 (PMC5168509; doi:10.1136/bmjopen-2015-010957)
Supplement: supplementary tables [file bmjopen-2015-010957supp_tables.pdf]

Supplementary Table S1: Sensitivity analysis: intention to treat analyses of the effect of AFLY5 intervention on primary and secondary outcomes 12 months post-intervention. Numbers vary by outcome as indicated in the table. In these analyses participants were only included for each outcome if they had a baseline and a follow-up measurement of that outcome.

**Outcome**	**Main comparison between the two groups (Intervention versus Control)**		
**Primary** / secondary	**Np**	**Difference in means or odds ratio (95%CI)**	**p-value**
**Continuous outcomes**			
**Time spent in MVPA (minutes per day)**	1000	3.05 (-1.33, 7.44)	0.17
**Time spent in sedentary behaviour (minutes per day)**	1000	2.21 (-8.28, 12.71)	0.68
**Servings of fruit and vegetables (number per day)**	1953	0.02 (-0.15, 0.19)	0.83
Time spent screen-viewing (minutes per day weekday)	1965	-10.53 (-26.1, 5.05)	0.19
Time spent screen-viewing (minutes per day Saturday)	1965	-17.3 (-33.71, -0.88)	0.04
Body mass index (z(sd)-score)	1563	0 (-0.05, 0.04)	0.95
Waist circumference (z(sd)-score)	1748	-0.03 (-0.12, 0.05)	0.47
Servings of snacks (number per day)	1953	-0.13 (-0.3, 0.04)	0.13
Servings of high fat foods (number per day)	1953	-0.13 (-0.25, 0)	0.04
Servings of high energy drinks (number per day)	1953	-0.21 (-0.4, -0.02)	0.03
**Binary outcomes**			
Generally overweight/obese	1563	0.83 (0.56, 1.22)	0.35
Centrally overweight/obese	1748	1.01 (0.73, 1.4)	0.93

Np: number of participants; MVPA: moderate or vigorous physical activity; CI: confidence interval
Outcomes in bold are primary outcomes ($p < 0.05$ indicates statistical significance); all others are secondary outcomes ($p < 0.01$ indicates statistical significance, after taking account of multiple testing).
All differences in means / odds ratios with their 95%CI have been estimated using a multi-level model to account for clustering (non-independence) among children from the same school. Multi-level multivariable linear regression was used for effects of the intervention on continuously measured outcomes and multi-level multivariable logistic regression was used for binary outcomes.
The following baseline / school stratifying covariables were included: age, gender, the baseline measure of the outcome under consideration, school involvement in other health promoting behaviours, school area level deprivation.
MVPA: moderate and vigorous physical activity (accelerometer assessed), SB: sedentary behaviour (accelerometer assessed), BMI: body mass index, WC: waist circumference, F&V fruit and vegetables.

Supplementary Table S2: Sensitivity analysis: intention to treat analyses of the effect of AFLY5 intervention on primary and secondary outcomes assessed 12 months post-intervention. In these analyses participants were only included for each outcome if they had a baseline and a follow-up measurement for all three primary outcomes. Numbers included are identical for the three primary outcomes (N = 757) but can vary by outcome for secondary outcomes (though none of these can be higher than 757) as indicated in the table.

**Outcome**	**Main comparison between the two groups (Intervention versus Control)**		
	**Np**	**Difference in means or odds ratio (95%CI)**	**p-value**
**Continuous outcomes**			
**Time spent in MVPA (minutes per day)**	757	1.28 (-3.22, 5.78)	0.58
**Time spent in sedentary behaviour (minutes per day)**	757	0.60 (-10.44, 11.63)	0.92
**Servings of fruit and vegetables (number per day)**	757	-0.13 (-0.34, 0.09)	0.26
Time spent screen-viewing (minutes per day weekday)	757	0.20 (-17.54, 17.94)	0.98
Time spent screen-viewing (minutes per day Saturday)	757	-8.46 (-28.49, 1.56)	0.41
Body mass index (z(sd)-score)	682	0.00 (-0.06, 0.07)	0.80
Waist circumference (z(sd)-score)	728	-0.01 (-0.12, 0.09)	0.90
Servings of snacks (number per day)	757	-0.13 (-0.38, 0.13)	0.33
Servings of high fat foods (number per day)	757	-0.13 (-0.33, 0.07)	0.19
Servings of high energy drinks (number per day)	757	-0.12 (-0.37, 0.12)	0.32
**Binary outcomes**			
Generally overweight/obese	680	1.09 (0.64, 1.85)	0.76
Centrally overweight/obese	728	11.35 (0.81, 2.23)	0.25

Np: number of participants; MVPA: moderate or vigorous physical activity; CI: confidence interval

Outcomes in bold are primary outcomes ($p < 0.05$ indicates statistical significance); all others are secondary outcomes ($p < 0.01$ indicates statistical significance, after taking account of multiple testing).

All differences in means / odds ratios with their 95%CI have been estimated using a multi-level model to account for clustering (non-independence) among children from the same school. Multi-level multivariable linear regression was used for effects of the intervention on continuously measured outcomes and multi-level multivariable logistic regression was used for binary outcomes.

The following baseline / school stratifying covariables were included: age, gender, the baseline measure of the outcome under consideration, school involvement in other health promoting behaviours, school area level deprivation.

MVPA: moderate and vigorous physical activity (accelerometer assessed), SB: sedentary behaviour (accelerometer assessed), BMI: body mass index, WC: waist circumference, F&V fruit and vegetables.
Missing baseline data for secondary outcomes (once those with missing baseline primary outcomes are excluded) were managed as in the main analyses.

Supplementary Table S3: Sensitivity analysis: intention to treat analyses of the effect of AFLY5 intervention on primary and secondary outcomes assessed 12 months post-intervention, with missing data for either baseline or follow-up measure of an outcome assumed to be 10% healthier than the average value in the study sample.

Outcome	Main comparison between the two groups (Intervention versus Control)		
	**Np**	**Difference in means or odds ratio (95%CI)**	**p-value**
**Continuous outcomes**			
**Time spent in MVPA (minutes per day)**	2052	0.74 (-1.59, 3.07)	0.53
**Time spent in sedentary behaviour (minutes per day)**	2052	1.78 (-4.63, 8.20)	0.59
**Servings of fruit and vegetables (number per day)**	2052	0.01 (-0.16, 0.17)	0.94
Time spent screen-viewing (minutes per day weekday)	2052	-10.74 (-26.30, 4.81)	0.18
Time spent screen-viewing (minutes per day Saturday)	2052	-16.03 (-32.82, 0.76)	0.06
Body mass index (z(sd)-score)	2052	0.01 (-0.04, 0.06)	0.70
Waist circumference (z(sd)-score)	2052	-0.02 (-0.11, 0.06)	0.56
Servings of snacks (number per day)	2052	-0.11 (-0.29, 0.06)	0.19
Servings of high fat foods (number per day)	2052	-0.12 (-0.25, 0.00)	0.05
Servings of high energy drinks (number per day)	2052	-0.20 (-0.39, -0.01)	0.04
**Binary outcomes**			
Generally overweight/obese	2052	0.98 (0.76, 1.26)	0.87
Centrally overweight/obese	2052	1.05 (0.77, 1.43)	0.78

Np: number of participants; MVPA: moderate or vigorous physical activity; CI: confidence interval

Outcomes in bold are primary outcomes ($p < 0.05$ indicates statistical significance); all others are secondary outcomes ($p < 0.01$ indicates statistical significance, after taking account of multiple testing).

All differences in means / odds ratios with their 95%CI have been estimated using a multi-level model to account for clustering (non-independence) among children from the same school. Multi-level multivariable linear regression was used for effects of the intervention on continuously measured outcomes and multi-level multivariable logistic regression was used for binary outcomes.

The following baseline / school stratifying covariables were included: age, gender, the baseline measure of the outcome under consideration, school involvement in other health promoting behaviours, school area level deprivation.

MVPA: moderate and vigorous physical activity (accelerometer assessed), SB: sedentary behaviour (accelerometer assessed), BMI: body mass index, WC: waist circumference, F&V fruit and vegetables.
In these analyses participants all participants are included (N = 2,221 (the number of participants recruited to the study). Missing baseline data is managed as in the main analyses and missing outcome data are imputed on the basis of those with missing data being 10% healthier than all participants in the study for a given outcome.

Supplementary Table S4: Sensitivity analysis: intention to treat analyses of the effect of AFLY5 intervention on primary and secondary outcomes assessed 12 months post-intervention, with missing data for either baseline or follow-up measure of an outcome assumed to be 10% less healthy than the average value in the study sample.

**Outcome**	**Main comparison between the two groups (Intervention versus Control)**		
	**Np**	**Difference in means or odds ratio (95%CI)**	**p-value**
**Continuous outcomes**			
**Time spent in MVPA (minutes per day)**	2052	1.04 (-1.18, 3.26)	0.36
**Time spent in sedentary behaviour (minutes per day)**	2052	-0.72 (-6.39, 4.95)	0.80
**Servings of fruit and vegetables (number per day)**	2052	0.01 (-0.16, 0.17)	0.94
Time spent screen-viewing (minutes per day weekday)	2052	-10.74 (-26.30,4.81)	0.18
Time spent screen-viewing (minutes per day Saturday)	2052	-16.03 (-32.82, 0.76)	0.06
Body mass index (z(sd)-score)	2052	0.01 (-0.04, 0.06)	0.70
Waist circumference (z(sd)-score)	2052	-0.02 (-0.11, 0.06)	0.56
Servings of snacks (number per day)	2052	-0.11 (-0.29, 0.06)	0.19
Servings of high fat foods (number per day)	2052	-0.12 (-0.25, 0.00)	0.05
Servings of high energy drinks (number per day)	2052	-0.20 (-0.39, -0.01)	0.04
**Binary outcomes**			
Generally overweight/obese	2052	0.98 (0.76, 1.26)	0.87
Centrally overweight/obese	2052	1.05 (0.77, 1.43)	0.78

Np: number of participants; MVPA: moderate or vigorous physical activity; CI: confidence interval

Outcomes in bold are primary outcomes ($p < 0.05$ indicates statistical significance); all others are secondary outcomes ($p < 0.01$ indicates statistical significance, after taking account of multiple testing).

All differences in means / odds ratios with their 95%CI have been estimated using a multi-level model to account for clustering (non-independence) among children from the same school. Multi-level multivariable linear regression was used for effects of the intervention on continuously measured outcomes and multi-level multivariable logistic regression was used for binary outcomes.

The following baseline / school stratifying covariables were included: age, gender, the baseline measure of the outcome under consideration, school involvement in other health promoting behaviours, school area level deprivation.

MVPA: moderate and vigorous physical activity (accelerometer assessed), SB: sedentary behaviour (accelerometer assessed), BMI: body mass index, WC: waist circumference, F&V fruit and vegetables.

In these analyses participants all participants are included (N = 2,221 (the number of participants recruited to the study). Missing baseline data is managed as in the main table and missing outcome data are imputed on the basis of those with missing data being 10% less healthy than all participants in the study for a given outcome.

Supplementary Table S5: Main intention to treat analyses of the effect of AFLY5 intervention on accelerometer-assessed outcomes during 3 valid days, separately for week and weekend days. Numbers vary by outcome as indicated in the table.

Outcome	Main comparison between the two groups (Intervention versus Control) on week days			Main comparison between the two groups (Intervention versus Control) on weekend days		
	**Np**	**Difference in means (95%CI)**	**p-value**	**Np**	**Difference in means (95%CI)**	**p-value**
**Time spent in MVPA (minutes per day)**	1627	2.47 (-1.37, 6.32)	0.21	972	3.26 (-3.62, 10.14)	0.35
**Time spent in sedentary behaviour (minutes per day)**	1627	1.87 (-8.51, 12.24)	0.72	972	3.07 (-10.91, 17.06)	0.67

Np: number of participants; MVPA: moderate or vigorous physical activity; CI: confidence interval

All differences in means with their 95%CI have been estimated using a multi-level model to account for clustering (non-independence) among children from the same school. Multi-level multivariable linear regression was used for effects of the intervention on continuously measured outcomes.

The following baseline / school stratifying covariables were included: age, gender, the baseline measure of the outcome under consideration, school involvement in other health promoting behaviours, school area level deprivation.

MVPA: moderate and vigorous physical activity (accelerometer assessed), SB: sedentary behaviour (accelerometer assessed).

In these analyses, participants were only included for each outcome if they had a follow-up measurement of that outcome. For partial missing baseline data we used an indicator variable as describe by White & Thompson,(1) which means for each outcome participants are included even if they do not have a baseline measurement.

Only participants included in the main analyses (i.e. with at least 3 valid days of accelerometer data) are included in this sensitivity analysis.

References

1. White IR, Thompson SG. Adjusting for partially missing baseline measurements in randomized trials. Stat Med. 2005;24(7):993-1007. Epub 2004/12/01.
